# The association between maternal characteristics and SARS-CoV-2 in pregnancy: a population-based registry study in Sweden and Norway

**DOI:** 10.1038/s41598-022-12395-y

**Published:** 2022-05-19

**Authors:** Anne K. Örtqvist, Maria C. Magnus, Jonas Söderling, Laura Oakley, Anne-Marie Nybo Andersen, Siri E. Håberg, Olof Stephansson

**Affiliations:** 1 Clinical Epidemiology Division, Department of Medicine, Karolinska Institutet, Karolinska Universitetssjukhuset, Solna, T2:02, 171 76 Stockholm, Sweden; 2 Department of Obstetrics and Gynecology, Visby County Hospital, Visby, Sweden; 3 Centre for Fertility and Health, Norwegian Institute of Public Health, Oslo, Norway; 4 Department of Non-Communicable Disease Epidemiology, London School of Hygiene and Tropical Medicine, London, UK; 5 Department of Public Health, University of Copenhagen, Copenhagen, Denmark; 6 Department of Women’s Health, Division of Obstetrics, Karolinska University Hospital, Stockholm, Sweden

**Keywords:** Epidemiology, Risk factors

## Abstract

The objectives of the current study were to identify risk factors for SARS-CoV-2 positivity, and to address how different testing strategies, choice of comparison group, and population background characteristics may influence observed associations. National registries data for 107,627 pregnant women in Sweden and 81,195 in Norway, were used to identify risk factors for SARS-CoV-2, separately for women under non-universal testing (testing by indication) and universal testing (testing of all pregnant women in contact with a delivery ward). We also investigated underlying characteristics associated with testing for SARS-CoV-2. Overall, 2.1% of pregnant women in Sweden and 1.1% in Norway were test-positive during the pandemic’s first 18 months. We show that the choice of test strategy for SARS-CoV-2 provided different associations with risk factors for the disease; for instance, women who were overweight, obese or had gestational diabetes had increased odds of being test-positive under non-universal testing, but not under universal testing. Nevertheless, a consistent pattern of association between being born in the Middle East and Africa and test-positivity was found independent of test strategy and in both countries. These women were also less likely to get tested. Our results are useful to consider for surveillance and clinical recommendations for pregnant women during the current and future pandemics.

## Introduction

Pregnant women are at higher risk for severe acute respiratory syndrome coronavirus 2 (SARS-CoV-2) infection causing corona virus disease 2019 (COVID-19)^1^. While pregnant and non-pregnant women seem to be at similar risk of SARS-CoV-2 test-positivity^2^, previous studies have shown that pregnant women with COVID-19 are at increased risk of hospitalization^1,2^, admission to intensive care unit^1,3^ and invasive interventions^1^, compared to non-pregnant women of reproductive age. There is also growing evidence of increased risks associated with maternal SARS-CoV-2 infection for fetuses and neonates, such as intrauterine transmission^4^, preterm birth^1,5,6^, neonatal morbidities^7^ and fetal death^5^.

Identifying risk factors associated with SARS-CoV-2 infection in pregnancy is important for surveillance and recommendations to pregnant women. A living systematic review and meta-analysis on COVID-19 in pregnancy by Allotey et al., has reported several risk factors for severe COVID-19 among pregnant women, including advanced maternal age, high body mass index (BMI), chronic hypertension, pre-existing diabetes and pregnancy-related disorders such as pre-eclampsia^1^. However, the meta-analysis of risk factors for SARS-CoV-2 positivity during pregnancy independent of disease severity, only found non-white ethnicity to be a risk factor^1^. Few previous studies have been population-based^5,8–10^, often limited to specific hospitals^11–14^ or regions^15^ or limited to the first wave of the pandemic^9^, and thus most studies predominantly include a small number of test-positive women^12,14,15^. Furthermore, different test strategies, such as non-universal testing where testing is performed by indication (for example, in the presence of symptoms) and thus excluding a large group of potential asymptomatic SARS-CoV-2 test-positive individuals, or using universal testing of all pregnant women (independent of symptoms), may strongly influence previous findings^1,16^. Under non-universal testing, difference in background characteristics, such as sociodemographic factors will also most likely affect the likelihood of getting tested or not, and thus affect who is included in such studies.

The objectives of the current study were to identify risk factors for SARS-CoV-2 positivity, and to address how different testing strategies, choice of comparison group, and population background characteristics may influence observed associations.

## Material and methods

### Study population and design

**Figure 1:**
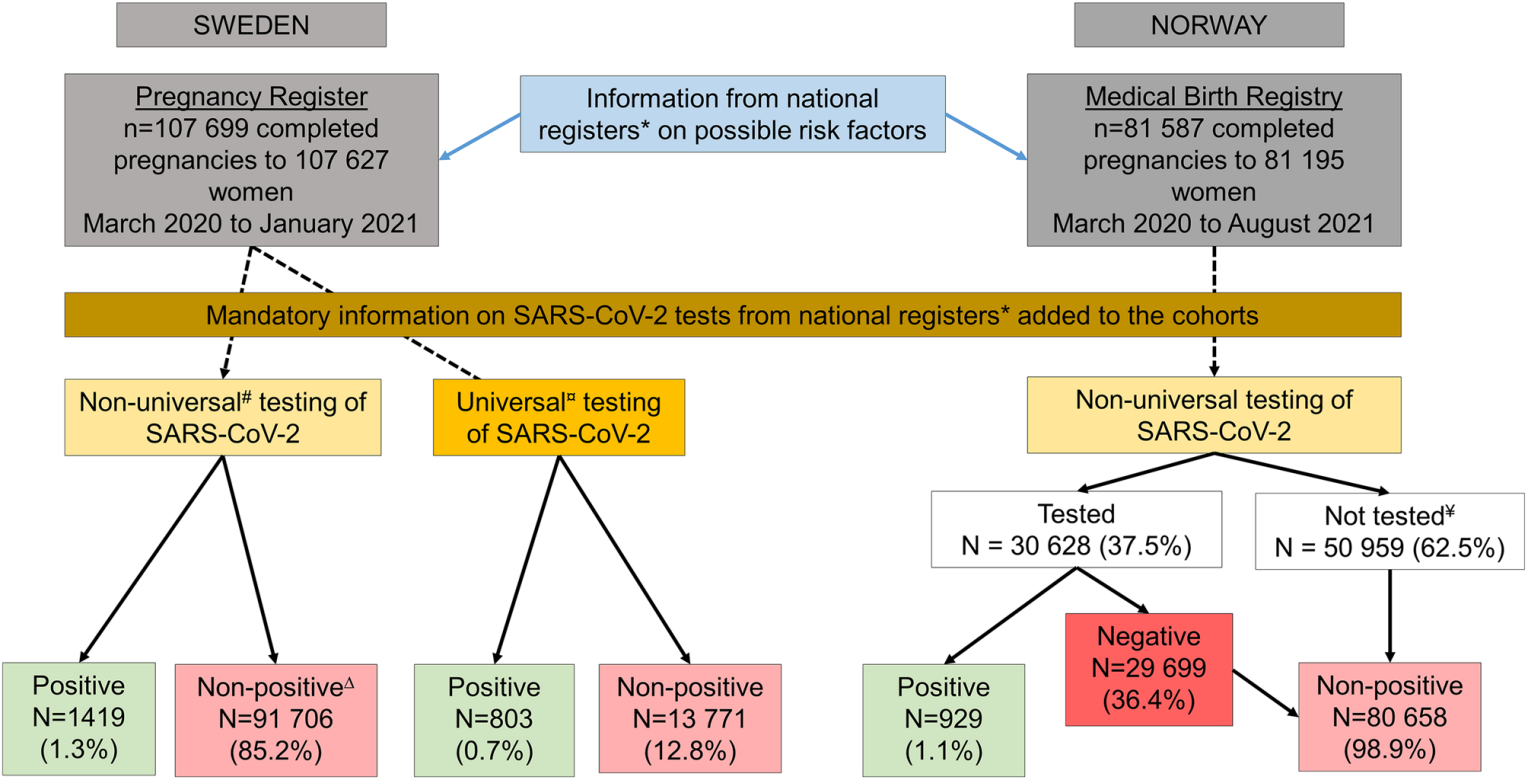
Flow chart of study populations under non-universal and universal testing in Sweden and Norway. *The Swedish data of deliveries was linked to the Total population register and Education register at Statistics Sweden and the National Register for Communicable Disease (SmiNet at the Public Health Agency. The Norwegian data of deliveries was linked to information from the National population register, educational information from Statistics Norway, and the Norwegian Surveillance System for Communicable Diseases. ^#^Non-universal testing mostly included symptomatic individuals, but it could also include individuals tested before and after travels, or after contact with other test-positive individuals and individuals subject to workplace testing. ^¤^Universal testing of all women admitted to the delivery ward or for other medical reasons requiring in-patient care were tested, independent of COVID-19 symptoms or not, was conducted in 23 of the 39 delivery hospitals covered by the Swedish Pregnancy Register. ^∆^As information on those with a negative test was not available for Swedish data, the comparison group in analysis of Swedish data included both those had been tested negative but also those not tested at all, i.e. non-positives. ^¥^Not tested for SARS-CoV-2.

This registry-based study included women registered with a delivery after 22 gestational weeks between March 2020 and January 2021 in the Swedish Pregnancy Register^17^ and between March 2020 and August 2021 in the Medical Birth Registry of Norway. The Swedish Pregnancy Register covers 94% of all births (live and stillbirths) after 22 completed gestational weeks in Sweden (18 of 21 regions), while the Medical Birth Registry in Norway includes all pregnancies ending in gestational week 12 or later. Both countries provide free health care during pregnancy. Unique personal identity numbers are assigned to all citizens in Sweden and Norway at birth or immigration enabling linkage of data across national registries. The Swedish data of deliveries was linked to the Total Population Register and Education Register at Statistics Sweden and the National Register for Communicable Disease (SmiNet) at the Public Health Agency. The Norwegian data of deliveries was linked to information from the National Population Register, educational information from Statistics Norway, and the Norwegian Surveillance System for Communicable Diseases (Fig. 1). The registers are described in more detail in the Supplementary Information.

### Testing strategies and reporting of SARS-CoV-2

In Sweden, SARS-CoV-2 was included in the Swedish Communicable Disease Act on 1st February, 2020, making all laboratory-confirmed polymerase chain reaction (PCR) cases of SARS-CoV-2 mandatory to report within 24 h to SmiNet at the Public Health Agency of Sweden^7,18^. Negative tests results are unfortunately not available on a national level. A non-universal population testing strategy was implemented including outpatient testing and contact tracing, starting in June 2020. This type of testing mostly included symptomatic individuals, but it could also include individuals tested before and after travels, or after contact with other test-positive individuals and individuals subject to workplace testing (e.g. healthcare workers)^18^. In 23 of the 39 delivery hospitals covered by the Swedish Pregnancy Register, universal testing of all women admitted for labor or pregnancy in-patient care, independent of their current and previous medical history and COVID-19 symptoms or not, were performed (Fig. 1). More information on the hospitals and dates for implementing universal testing can be found in the Supporting Information (Table S1).

In Norway, mandatory registration of all PCR tests for SARS-CoV-2 was implemented in the Norwegian Surveillance System for Communicable Diseases on 31st January, 2020. As Norway has not had any universal testing of pregnant or delivering women, a non-universal testing strategy was operating throughout the study period. Testing was predominantly conducted on the basis of symptoms to confirm or exclude SARS-CoV-2 infection, with some additional testing conducted for particular reasons such as contact with infected persons, or mandatory testing due to travel or work. Information was available on all conducted tests, regardless of positive or negative result, providing the opportunity to evaluate characteristics of all women being tested in Norway, and to compare risk factors for test-positivity among those tested (Fig. 1).

For both Sweden and Norway, women were defined as test-positive during any time in pregnancy if they had a positive PCR test for SARS-CoV-2 between the estimated start of pregnancy and the date of delivery. The start of pregnancy was estimated using the date of delivery minus the estimated pregnancy length, as defined from routine ultrasound scans performed in 98% of pregnancies, or otherwise from last menstrual period. Number of days from a positive test to delivery was calculated based on date of delivery minus the test date. If several positive tests for SARS-CoV-2 were registered (n = 10), the first was used in the statistical analyses.

No one in the Swedish population had been vaccinated against COVID-19 during the study period, while 428 (0.5%) women in the Norwegian population were vaccinated.

### Investigated characteristics

Background characteristics potentially associated with SARS-CoV-2 test-positivity were identified from previous studies and are described in the Supporting Information. These were divided into three groups: (1) maternal background characteristics including age, parity, early pregnancy BMI, education level, birth region, smoking status in early pregnancy, work situation, cohabitation with partner and number of persons in the household (only Sweden); (2) pre-existing co-morbidities including chronic hypertension, diabetes, lung disease/asthma, chronic kidney disease, cardiovascular disease, prior thrombosis; and (3) pregnancy related factors such as multiple pregnancy, pre-eclampsia/HELLP syndrome (Hemolysis, Elevated Liver Enzymes, Low platelet counts)/eclampsia, gestational diabetes, and whether the pregnancy was conceived by in vitro fertilization (IVF).

### Statistical analyses

Analyses were conducted within each country separately according to a standardized study protocol. For women in Sweden, the associations between maternal characteristics, pre-existing co-morbidities and pregnancy related factors with SARS-CoV-2 test-positivity, were analyzed with crude and adjusted logistic regression models separately for those under universal and non-universal testing. The comparison group (non-positive) included women who tested negative and those who were not tested. For women in Norway, crude and adjusted logistic regression analysis for underlying characteristics and test-positivity was performed. First, the Norwegian data was analyzed in the same way as the Swedish data and compared test-positive women to all other pregnant women in Norway (i.e. those not tested or test-negative). Secondly, to investigate the impact of testing strategy, risk factors in the test-positive women were compared to the test-negative women. Finally, characteristics associated with the likelihood of being tested for SARS-CoV-2 during pregnancy was examined in Norwegian data. All adjusted analyses included maternal age, education level and birth region.

All methods were performed in accordance with relevant guidelines and regulations. Statistical analyses were performed with SAS version 9.4 (Sweden) and Stata version 16 (Norway).

### Ethical approval

This study was approved by the Swedish Ethical Review Authority (approval numbers: dnr 2020-01499, dnr 2020-02468, dnr 2021-00274) and Regional Committee for Medical and Health Research Ethics of South/East Norway (#141135). Each committee provided a waiver of consent for participants.

## Results

In total, 107,627 women with 107,699 completed pregnancies in Sweden, and 81,195 women with 81,587 completed pregnancies in Norway were included in the study (Fig. 1). In Sweden, 2.1% (n = 2222) of pregnant women tested positive for SARS-CoV-2 during the study period, of which 36% were identified through universal testing and 64% in non-universal testing. In Norway, 1.1% (n = 929) of pregnant women tested positive during the study period. Pregnant women in Sweden were tested 39 days (median) before delivery (interquartile range (IQR) 8; 88), with a median of 26 days (IQR 1; 65) for those under universal testing and 46 days (IQR 13; 101) for those under non-universal testing. In Norway, pregnant women were tested approximately 98 days (median) before delivery (IQR 44; 161).

### Characteristics of SARS-CoV-2 test-positive pregnant women in Sweden

Table 1 shows the characteristics of test-positive pregnant women in Sweden. Being overweight or obese (adjusted Odds Ratio (aOR) range 1.21–1.54, and having gestational diabetes (aOR 1.67, 95% Confidence Interval (CI) 1.38–2.01), was associated with increased odds of test-positivity in pregnant women under non-universal testing, but not for women under universal testing. Women born in other European countries (aOR 1.28, 95% CI 1.06–1.54) and in the Middle East or Africa (aOR 1.79, 95% CI 1.56–2.05) were more likely to be test-positive than those born in Scandinavia in analyses of women under non-universal testing. The association remained for women born in the Middle East or Africa (aOR 1.68, 95% CI 1.41–2.00) in analyses of women under universal testing. Pregnant women living with five or more individuals in the household were more likely to be test-positive in analyses of those both under non-universal (aOR 1.36, 95% CI 1.15–1.62) and universal testing (aOR 1.36, 95% CI 1.08–1.70). Smoking was associated with decreased odds of test-positivity in women under non-universal testing (aOR 0.46, 95% CI 0.31–0.70), although this association was not observed in analyses of women under universal testing (aOR 1.06, 95% CI 0.72–1.56).

**Table 1:** Unadjusted and adjusted logistic regression models for the likelihood of SARS-CoV-2 test-positivity compared to non-positive (test-negative and not tested) under non-universal testing and under universal testing in Sweden, in relation to characteristics.

Characteristics	Non-universal testing				Universal testing			
	Test-positive n = 1419	Non-Positive n = 91,706	Unadjusted model	Adjusted for age, birth region and education*	Test-positive n = 803	Non-Positive n = 13,771	Unadjusted model	Adjusted for age, birth region and education*
	n (%)	n (%)	OR (95% CI)	OR (95% CI)	n (%)	n (%)	OR (95% CI)	OR (95% CI)
**Background**								
Age (years)								
< 25	128 (9.0)	7993 (8.7)	1.07 (0.88–1.30)	1.00 (0.81–1.23)	69 (8.6)	1227 (8.9)	0.96 (0.73–1.25)	0.85 (0.64–1.12)
25–29	436 (30.7)	28,258 (30.8)	1.03 (0.91–1.17)	1.03 (0.91–1.18)	235 (29.3)	3846 (27.9)	1.04 (0.87–1.24)	1.01 (0.85–1.21)
30–34	516 (36.4)	34,489 (37.6)	Reference	Reference	309 (38.5)	5250 (38.1)	Reference	Reference
≥ 35	339 (23.9)	20,966 (22.9)	1.08 (0.94–1.24)	1.05 (0.91–1.20)	190 (23.7)	3448 (25.0)	0.94 (0.78–1.13)	0.90 (0.75–1.09)
Parity								
0	625 (44.0)	39,481 (43.1)	Reference	Reference	332 (41.3)	6062 (44.0)	Reference	Reference
1	472 (33.3)	34,294 (37.4)	0.87 (0.77–0.98)	0.85 (0.75–0.96)	264 (32.9)	4843 (35.2)	1.00 (0.84–1.18)	0.99 (0.83–1.17)
2	199 (14.0)	12,208 (13.3)	1.03 (0.88–1.21)	0.94 (0.79–1.11)	128 (15.9)	1874 (13.6)	1.25 (1.01–1.54)	1.17 (0.93–1.46)
≥ 3	123 (8.7)	5723 (6.2)	1.36 (1.12–1.65)	1.11 (0.89–1.39)	79 (9.8)	992 (7.2)	1.45 (1.13–1.88)	1.26 (0.94–1.69)
BMI								
< 18.5	23 (1.7)	2072 (2.4)	0.81 (0.54–1.24)	0.79 (0.52–1.20)	9 (1.2)	325 (2.5)	0.50 (0.26–0.97)	0.47 (0.24–0.93)
18.5–< 25	653 (47.8)	47,889 (54.5)	Reference	Reference	389 (50.1)	7005 (53.0)	Reference	Reference
25–< 30	408 (29.9)	23,917 (27.2)	1.25 (1.10–1.42)	1.21 (1.07–1.37)	229 (29.5)	3639 (27.5)	1.13 (0.96–1.34)	1.09 (0.92–1.29)
30–< 35	188 (13.8)	9516 (10.8)	1.45 (1.23–1.71)	1.39 (1.18–1.64)	102 (13.1)	1542 (11.7)	1.19 (0.95–1.49)	1.12 (0.89–1.40)
≥ 35	93 (6.8)	4435 (5.0)	1.54 (1.24–1.92)	1.54 (1.24–1.93)	48 (6.2)	713 (5.4)	1.21 (0.89–1.65)	1.16 (0.84–1.58)
* Missing*	54	3877			26	547		
Educational level (years)								
≤ 9	150 (11.1)	8426 (9.6)	1.18 (0.99–1.41)	0.93 (0.77–1.13)	109 (14.3)	1419 (10.9)	1.44 (1.15–1.80)	1.12 (0.88–1.43)
10–12	530 (39.1)	34,972 (39.7)	1.00 (0.90–1.13)	0.99 (0.88–1.11)	311 (40.7)	5200 (39.8)	1.12 (0.96–1.31)	1.06 (0.90–1.25)
> 12	676 (49.9)	44,792 (50.8)	Reference	Reference	344 (45.0)	6446 (49.3)	Reference	Reference
* Missing*	63	3516			39	706		
Birth region								
Scandinavia^#^	887 (62.6)	65, 221 (71.1)	Reference	Reference	473 (58.9)	9084 (66.0)	Reference	Reference
Other European country	135 (9.5)	7830 (8.5)	1.27 (1.06–1.52)	1.28 (1.06–1.54)	61 (7.6)	1140 (8.3)	1.03 (0.78–1.35)	1.07 (0.81–1.41)
Middle East/Africa	342 (24.2)	14,493 (15.8)	1.74 (1.53–1.97)	1.79 (1.56–2.05)	236 (29.4)	2713 (19.7)	1.67 (1.42–1.96)	1.68 (1.41–2.00)
Other	52 (3.7)	4123 (4.5)	0.93 (0.70–1.23)	0.93 (0.70–1.24)	33 (4.1)	824 (6.0)	0.77 (0.54–1.10)	0.80 (0.56–1.16)
Missing	3	39			0	10		
Smoking status								
Smoker, yes	24 (1.8)	3310 (3.7)	0.46 (0.31–0.69)	0.46 (0.31–0.70)	29 (3.7)	463 (3.4)	1.07 (0.73–1.57)	1.06 (0.72–1.56)
*Missing*	50	3319			16	315		
Work situation								
Employed/maternity leave/student	1180 (91.6)	77,697 (92.7)	Reference	Reference	648 (90.8)	11,247 (90.9)	Reference	Reference
Unemployed/sick leave/other	108 (8.4)	6154 (7.3)	1.16 (0.95–1.41)	1.03 (0.84–1.26)	66 (9.2)	1123 (9.1)	1.02 (0.79–1.32)	0.92 (0.71–1.21)
* Missing*	131	7855			89	1 401		
Co-habits with partner								
No	100 (7.2)	7073 (7.9)	0.91 (0.74–1.11)	0.83 (0.68–1.02)	68 (8.5)	1225 (9.0)	0.94 (0.73–1.21)	0.84 (0.65–1.09)
* Missing*	26	1729			6	196		
Number of persons in the household								
1	94 (6.7)	4952 (5.5)	1.32 (1.05–1.65)	1.31 (1.05–1.64)	56 (7.1)	869 (6.4)	1.15 (0.85–1.54)	1.12 (0.83–1.52)
2	437 (31.2)	30,306 (33.4)	Reference	Reference	247 (31.3)	4390 (32.4)	Reference	Reference
3	409 (29.2)	31,556 (34.7)	0.90 (0.78–1.03)	0.88 (0.77–1.01)	219 (27.8)	4425 (32.7)	0.88 (0.73–1.06)	0.87 (0.72–1.05)
4	220 (15.7)	13,379 (14.7)	1.14 (0.97–1.34)	1.06 (0.90–1.26)	108 (13.7)	2053 (15.2)	0.93 (0.74–1.18)	0.87 (0.69–1.10)
≥ 5	240 (17.1)	10,651 (11.7)	1.56 (1.33–1.83)	1.36 (1.15–1.62)	159 (20.2)	1800 (13.3)	1.57 (1.28–1.93)	1.36 (1.08–1.70)
* Missing*	19	862			14	234		
**Pre-existing co-morbidities**								
Chronic hypertension	6 (0.4)	441 (0.5)	0.88 (0.39–1.97)	0.87 (0.39–1.95)	7 (0.9)	100 (0.7)	1.20 (0.56–2.60)	1.18 (0.55–2.56)
Diabetes	18 (1.3)	1043 (1.1)	1.12 (0.70–1.78)	1.08 (0.68–1.73)	16 (2.0)	206 (1.5)	1.34 (0.80–2.24)	1.32 (0.79–2.21)
Lung disease/asthma	107 (7.5)	6341 (6.9)	1.10 (0.90–1.34)	1.18 (0.97–1.44)	51 (6.4)	1024 (7.4)	0.84 (0.63–1.13)	0.90 (0.67–1.21)
Chronic kidney disease	6 (0.4)	360 (0.4)	1.08 (0.48–2.42)	1.07 (0.48–2.40)	2 (0.2)	63 (0.5)	0.54 (0.13–2.22)	0.55 (0.13–2.27)
Cardiovascular disease	17 (1.2)	1420 (1.5)	0.77 (0.48–1.25)	0.78 (0.48–1.26)	12 (1.5)	303 (2.2)	0.67 (0.38–1.21)	0.71 (0.40–1.27)
Prior thrombosis	12 (0.8)	782 (0.9)	0.99 (0.56–1.76)	1.05 (0.59–1.86)	9 (1.1)	141 (1.0)	1.10 (0.56–2.16)	1.11 (0.56–2.20)
Composite of the above	157 (11.1)	9600 (10.5)	1.06 (0.90–1.26)	1.12 (0.95–1.32)	89 (11.1)	1636 (11.9)	0.92 (0.74–1.16)	0.97 (0.77–1.22)
**Pregnancy related factors**								
Multiple pregnancy	21 (1.5)	1283 (1.4)	1.06 (0.69–1.64)	1.06 (0.68–1.63)	16 (2.0)	210 (1.5)	1.31 (0.79–2.19)	1.33 (0.79–2.22)
Pre-eclampsia, HELLP and eclampsia	57 (4.0)	3075 (3.4)	1.21 (0.92–1.58)	1.27 (0.97–1.65)	31 (3.9)	606 (4.4)	0.87 (0.60–1.26)	0.91 (0.63–1.32)
Gestational diabetes	128 (9.0)	4780 (5.2)	1.80 (1.50–2.17)	1.67 (1.38–2.01)	44 (5.5)	868 (6.3)	0.86 (0.63–1.18)	0.81 (0.59–1.11)
In vitro fertilization (IVF)	64 (4.5)	4441 (4.8)	0.93 (0.72–1.19)	0.95 (0.74–1.23)	27 (3.4)	653 (4.7)	0.70 (0.47–1.03)	0.75 (0.51–1.12)

*For the association between age and tested, the adjusted model included the covariate education and birth region. For the association between education and tested, the adjusted model included the covariates age and birth region. For the association between birth region and tested, the adjusted model included the covariates age and education. ^#^Sweden, Norway, Denmark.

### Characteristics of SARS-CoV-2 test-positive pregnant women in Norway

Table 2 shows the characteristics of pregnant women in Norway in the three groups: (1) Women who tested positive, (2) the combined group of test-negative and not tested women (i.e. non-positive), and (3) the women who tested negative. Some characteristics were consistent when comparing women with test-positivity to the combined group and to those who tested negative. Women with ≤ 12 years of education were more likely to be test-positive (≤ 9 years: aOR range 1.41–1.73; 10–12 years: aOR range 1.29–1.52). Moreover, compared to women born in Scandinavia, women from other European countries (aOR range 2.13–2.75), the Middle East or Africa (aOR range 4.66–5.95) and other countries (aOR range 1.83–2.22) had consistently higher odds of test-positivity. In analyses of pregnancy related factors, women with pre-eclampsia/HELLLP/eclampsia were less likely to be test-positive (aOR range 0.50–0.54) and women with gestational diabetes had increased odds of test-positivity in unadjusted analyses but not in adjusted analyses.

**Table 2:** Unadjusted and adjusted logistic regression models for the likelihood of SARS-CoV-2 test-positivity compared to non-positive (negative and not tested) and test-negative pregnant women in Norway, in relation to characteristics.

Characteristics	Test-positive n = 929^¤^	Non-positive n = 80,658	Test-negative n = 29,699	Test-positive vs. non-positive		Test-positive vs. test-negative	
				Unadjusted model	Adjusted for age, birth region and education*	Unadjusted model	Adjusted for age, birth region and education*
	n (%)	n (%)	n (%)	OR (95%CI)	OR (95%CI)	OR (95%CI)	OR (95%CI)
**Background**							
Age (years)							
< 25	71 (7.6)	6362 (7.9)	1948 (6.6)	0.94 (0.73–1.22)	0.76 (0.58–0.99)	1.20 (0.92–1.55)	0.82 (0.63–1.08)
25–29	277 (29.8)	24,407 (30.3)	8628 (29.1)	0.96 (0.82–1.12)	0.93 (0.80–1.09)	1.06 (0.90–1.23)	0.98 (0.83–1.15)
30–34	378 (40.7)	31,980 (39.7)	12,430 (41.9)	Reference	Reference	Reference	Reference
≥ 35	203 (21.9)	17,909 (22.2)	6693 (22.5)	0.96 (0.81–1.14)	0.86 (0.72–1.02)	1.00 (0.84–1.19)	0.85 (0.72–1.02)
Parity							
0	329 (35.4)	34,081 (42.3)	11,407 (38.4)	Reference	Reference	Reference	Reference
1	340 (36.6)	30,690 (38.1)	12,597 (42.4)	1.15 (0.99–1.33)	1.13 (0.96–1.32)	0.94 (0.80–1.09)	0.97 (0.83–1.14)
2	166 (17.3)	11,548 (14.3)	4347 (14.6)	1.44 (1.19–1.75)	1.27 (1.04–1.55)	1.28 (1.06–1.56)	1.16 (0.95–1.43)
≥ 3	99 (10.7)	4339 (5.4)	1348 (4.5)	2.36 (1.88–2.97)	1.46 (1.13–1.88)	2.54 (2.01–3.21)	1.43 (1.10–1.88)
BMI							
< 18.5	23 (2.7)	2448 (3.3)	811 (2.9)	0.87 (0.57–1.32)	0.72 (0.47–1.10)	0.98 (0.64–1.49)	0.76 (0.49–1.17)
18.5–< 25	472 (55.3)	43,848 (58.6)	16,256 (58.9)	Reference	Reference	Reference	Reference
25–< 30	232 (27.2)	17,800 (23.8)	6543 (23.7)	1.21 (1.03–1.42)	1.12 (0.95–1.31)	1.22 (1.04–1.43)	1.09 (0.93–1.29)
30–< 35	90 (10.5)	7145 (9.6)	2629 (9.5)	1.17 (0.93–1.47)	1.09 (0.86–1.37)	1.18 (0.94–1.48)	1.03 (0.61–1.22)
≥ 35	37 (4.3)	3557 (4.8)	1343 (4.9)	0.97 (0.69–1.35)	0.95 (0.68–1.34)	0.95 (0.68–1.33)	0.86 (0.83–1.38)
* Missing*	75	5860	2117				
Educational level (years)							
≤ 9	188 (24.6)	10,580 (14.4)	3217 (11.5)	2.12 (1.78–2.53)	1.41 (1.16–1.71)	2.86 (2.39–3.42)	1.73 (1.42–2.12)
10–12	179 (23.4)	15,612 (21.1)	5243 (18.7)	1.37 (1.15–1.63)	1.29 (1.08–1.54)	1.67 (1.40–2.00)	1.52 (1.27–1.83)
> 12*)*	398 (52.0)	47,494 (64.4)	19,505 (69.7)	Reference	Reference	Reference	Reference
* Missing*	164	6972	1734				
Birth region							
Scandinavia^#^	461 (49.7)	60,185 (74.7)	23,867 (81.4)	Reference	Reference	Reference	Reference
Other European c ountry	153 (16.5)	8883 (12.1)	2506 (8.4)	2.25 (1.87–2.70)	2.13 (1.75–2.60)	3.16 (2.62–3.81)	2.75 (2.25–3.36)
Middle East/Africa	224 (24.1)	5454 (6.8)	1450 (4.9)	5.36 (4.56–6.30)	4.66 (2.86–5.63)	8.00 (6.76–9.47)	5.95 (4.90–7.23)
Other	90 (9.8)	6047 (7.5)	1867 (6.3)	1.94 (1.55–2.44)	1.83 (1.45–2.32)	2.52 (2.01–3.18)	2.22 (1.75–2.82)
* Missing*	1	89	0				
Smoking status							
Smoker, yes	52 (6.3)	4175 (5.8)	1352 (5.1)	1.10 (0.83–1.46)	1.01 (0.76–1.35)	1.26 (0.95–1.68)	1.00 (0.74–1.34)
* Missing*	106	8205	3090				
Work situation							
Employed	527 (72.6)	58,156 (84.1)	22,378 (87.2)	Reference	Reference	Reference	Reference
Unemployed	199 (27.4)	10,999 (15.9)	3274 (12.8)	2.00 (1.69–2.35)	1.16 (0.96–1.40)	2.58 (2.18–3.05)	1.30 (1.08–1.58)
*Missing*	203	11,503	4047				
Co-habits with partner							
No	52 (5.7)	3213 (4.0)	1068 (3.7)	1.44 (1.09–1.91)	0.96 (0.72–1.28)	1.60 (1.20–2.13)	0.88 (0.65–1.19)
* Missing*	21	1234	490				
**Pre-existing co-morbidities**							
Chronic hypertension	3 (0.3)	462 (0.6)	184 (0.6)	0.56 (0.18–1.75)	0.53 (0.17–1.66)	0.52 (0.17–1.63)	0.44 (0.14–1.40)
Diabetes	8 (0.9)	572 (0.7)	215 (0.7)	1.22 (0.60–2.45)	1.35 (0.62–2.54)	1.19 (0.59–2.42)	1.09 (0.53–2.26)
Lung disease/asthma	52 (5.6)	5142 (6.4)	2048 (6.9)	0.87 (0.66–1.15)	0.86 (0.51–1.46)	0.80 (0.60–1.06)	0.98 (0.73–1.30)
Chronic kidney disease	1 (0.1)	472 (0.6)	178 (0.6)	–	–	–	–
Cardiovascular disease	0 (–)	461 (0.6)	173 (0.6)	–	–	–	–
Prior thrombosis	0 (–)	166 (0.2)	56 (0.2)	–	–	–	–
Composite of the above	61 (6.6)	6982 (8.7)	2738 (9.2)	0.74 (0.57–0.96)	0.87 (0.67–1.14)	0.69 (0.53–0.90)	0.80 (0.62–1.05)
**Pregnancy related factors**							
Multiple pregnancy	12 (1.3)	1071 (1.3)	427 (1.4)	0.97 (0.55–1.72)	0.97 (0.55–1.72)	0.90 (0.50–1.60)	0.88 (0.49–1.58)
Pre-eclampsia, HELLP and eclampsia	13 (1.4)	2215 (2.8)	842 (2.8)	0.50 (0.29–0.87)	0.54 (0.31–0.93)	0.49 (0.28–0.84)	0.50 (0.29–0.88)
Gestational diabetes	77 (8.3)	4758 (5.9)	1731 (5.8)	1.44 (1.14–1.82)	1.13 (0.89–1.43)	1.46 (1.15–1.85)	1.09 (0.85–1.39)
In vitro fertilization (IVF)	28 (3.0)	3819 (4.7)	1329 (4.5)	0.63 (0.43–0.91)	0.72 (0.49–1.06)	0.66 (0.45–0.97)	0.80 (0.54–1.17)

^¤^8 of 929 (0.9%) positive women had been vaccinated during the study period. *For the association between age and tested, the adjusted model included the covariate education and birth region. For the association between education and tested, the adjusted model included the covariates age and birth region. For the association between birth region and tested, the adjusted model included the covariates age and education. ^#^Sweden, Norway, Denmark.

Some estimates for test-positivity varied with comparison group. The probability of test-positivity increased with increasing number of previous births (parity), although significant for two or more births (aOR range 1.27–1.46) compared to the combined group and with three or more births (aOR 1.43, 95% CI 1.10–1.88) when compared to those who tested negative. Being unemployed was associated with increased odds of test-positivity when compared to women who tested negative (aOR 1.30, 95% CI 1.08–1.58), but not when compared to the combined group of non-tested and test-negative women (aOR 1.16, 95% CI 0.96–1.40).

### Characteristics of pregnant women tested for SARS-CoV-2 in Norway

Table 3 displays the characteristics related to the likelihood of being tested for SARS-CoV-2 in Norway. We found that having given birth one or more times previously (parity) was associated with increased odds of getting tested for SARS-CoV-2 (aOR range 1.07–1.38). Similarly, there was an indication that women with a BMI of 35 or more were more likely to get tested (aOR 1.07, 95% CI 1.00–1.15), as were women with asthma (aOR 1.09, 95% CI 1.03–1.16), gestational diabetes (aOR 1.07, 95% CI 1.01–1.14), any pre-existing co-morbidity (aOR 1.07, 95% CI 1.01–1.12) and women with multiple pregnancies (aOR 1.13, 95% CI 1.00–1.28). In contrast, women younger than 30 and above 35 years of age (aOR range 0.87–0.93), or with lower education level (≤ 12 years) (aOR range 0.71–0.76), those born in other countries than Scandinavia (aOR range 0.67–0.76), who were unemployed (aOR 0.88, 95% CI 0.84–0.92) or pregnant by IVF (aOR 0.84, 95% CI 0.77–0.92), were less likely to get tested. In crude analyses, smokers were less likely to get tested (aOR 0.82, 95% CI 0.77–0.88), but after adjustment for age, education level and birth region, the association was no longer significant (aOR 0.94, 95% CI 0.88–1.01).

**Table 3:** Unadjusted and adjusted logistic regression models for the likelihood of being tested (positive or negative) compared to not tested for SARS-CoV-2 in pregnant women in Norway in relation to characteristics.

Characteristics, n (%)	Tested n = 30,628 (24.6%)	Not tested n = 50,959 (75.4%)	Unadjusted model OR (95%CI)	Adjusted for age, birth region and education* OR (95%CI)
**Background**				
Age (years)				
< 25	2019 (6.6)	4414 (8.7)	0.70 (0.66–0.74)	0.87 (0.80–0.98)
25–29	8905 (29.1)	15,779 (31.0)	0.86 (0.83–0.89)	0.93 (0.88–0.98)
30–34	12,808 (41.8)	19,550 (38.4)	Reference	Reference
≥ 35y	6896 (22.5)	11,216 (22.0)	0.94 (0.90–0.97)	0.92 (0.87–0.98)
Parity				
0	11,736 (38.3)	22,674 (44.5)	Reference	Reference
1	12,937 (42.2)	18,093 (35.5)	1.38 (1.34–1.43)	1.38 (1.33–1.42)
2	4508 (14.7)	7201 (14.1)	1.21 (1.16–1.26)	1.23 (1.17–1.29)
≥ 3	1447 (4.7)	2991 (5.9)	0.93 (0.87–1.00)	1.07 (0.99–1.14)
BMI				
< 18.5	834 (2.9)	1637 (3.5)	0.84 (0.77–0.92)	0.93 (0.86–1.02)
18.5–< 25	16,728 (58.8)	27,592 (58.4)	Reference	Reference
25–< 30	6775 (23.8)	11,257 (23.8)	0.99 (0.96–1.03)	1.02 (0.98–1.05)
30–< 35	2719 (9.6)	4516 (9.6)	0.99 (0.96–1.10)	1.03 (0.98–1.08)
≥ 35	1380 (4.9)	2214 (4.7)	1.03 (0.96–1.10)	1.07 (1.00–1.15)
*Missing*	2192	3743		
Educational level (years)				
≤ 9	3405 (11.9)	7363 (16.1)	0.65 (0.62–0.70)	0.71 (0.68–0.75)
10–12	5422 (18.9)	10,369 (22.7)	0.74 (0.71–0.76)	0.76 (0.73–0.78)
> 12	19,903 (69.3)	27,989 (61.2)	Reference	Reference
*Missing*	1898	5238		
Birth region				
Scandinavia^#^	24,337 (79.5)	36,309 (71.3)	Reference	Reference
Other European country	2659 (8.7)	6377 (12.5)	0.62 (0.59–0.65)	0.67 (0.64–0.71)
Middle East/Africa	1674 (5.5)	4004 (7.9)	0.62 (0.59–0.67)	0.76 (0.71–0.81)
Other	1936 (6.3)	4201 (8.3)	0.69 (0.65–0.73)	0.74 (0.69–0.78)
* Missing*	22	68		
Smoking status				
Smoker	1404 (5.1)	2823 (6.2)	0.82 (0.77–0.88)	0.94 (0.88–1.01)
* Missing*	3196	5115		
Work situation				
Employed	22,905 (86.8)	35,778 (82.2)	Reference	Reference
Unemployed	3473 (13.2)	7725 (17.8)	0.70 (0.67–0.73)	0.88 (0.84–0.92)
*Missing*	4250	7456		
Co-habits with partner				
No	1120 (3.7)	2145 (4.3)	0.86 (0.80–0.93)	0.99 (0.92–1.07)
* Missing*	511	855		
**Pre-existing co-morbidities**				
Chronic hypertension	187 (0.6)	278 (0.6)	1.12 (0.93–1.35)	1.13 (0.93–1.36)
Diabetes	223 (0.7)	357 (0.7)	1.04 (0.88–1.23)	1.04 (0.88–1.23)
Lung disease/asthma	2100 (6.9)	3094 (6.1)	1.14 (1.08–1.21)	1.09 (1.03–1.16)
Chronic kidney disease	179 (0.6)	294 (0.6)	1.01 (0.84–1.22)	1.01 (0.84–1.22)
Cardiovascular disease	173 (0.5)	288 (0.6)	1.00 (0.83–1.21)	0.93 (0.77–1.13)
Prior thrombosis	56 (0.2)	110 (0.2)	0.85 (0.61–1.17)	0.79 (0.57–1.10)
Composite of the above	2799 (9.1)	4244 (8.3)	1.11 (1.05–1.16)	1.07 (1.01–1.12)
**Pregnancy related factors**				
Multiple pregnancy	439 (1.4)	644 (1.3)	1.14 (1.01–1.28)	1.13 (1.00–1.28)
Pre-eclampsia, HELLP and eclampsia	855 (2.8)	1373 (2.7)	1.04 (0.95–1.13)	1.03 (0.94–1.12)
Gestational diabetes	1808 (5.9)	3027 (5.9)	0.99 (0.94–1.05)	1.07 (1.01–1.14)
In vitro fertilization (IVF)	1357 (4.4)	2490 (4.9)	0.90 (0.84–0.97)	0.82 (0.76–0.88)

*For the association between age and tested, the adjusted model included the covariate education and birth region. For the association between education and tested, the adjusted model included the covariates age and birth region. For the association between birth region and tested, the adjusted model included the covariates age and education. ^#^Sweden, Norway, Denmark.

## Discussion

In this registry-based study including almost all pregnancies in Sweden and Norway during the first 18 months of the pandemic, we found that associations between maternal characteristics, pre-existing co-morbidities and pregnancy related factors with SARS-CoV-2 test-positivity differed according testing strategy and comparison groups. Only being born outside Scandinavia was robustly associated with test-positivity regardless of testing strategy or comparison group in both countries, while associations with other characteristics varied with comparison group or attenuated with adjustments. Similarly, the likelihood of being tested varied with several characteristics, such as having pre-existing co-morbidities, educational level or region of birth. Differences in comparison groups and testing may influence the interpretation of risk factors associated with SARS-CoV-2 during pregnancy, and consequently lead to inaccurate clinical recommendations for pregnant women.

It has been suggested that advanced maternal age is associated with severe COVID-19, but not with SARS-CoV-2 test-positivity per se^1^. Two recent studies have on the contrary suggested that younger age^13^ is associated with increased odds and older age^12^ with reduced odds of SARS-CoV-2 test-positivity. Our results indicate no associations between test-positivity and age. However, younger and older women were less likely to get tested than those 30–34 years, which may have resulted in lower representation of younger and older women in the analyses.

Parity can be regarded as a proxy for having older children in the household. Previous studies have not shown clear associations between parity and test-positivity^1^, which is supported by Swedish data. In Norway, there was a positive association with multiparity. We also found that, in Norway, multiparous women were more likely to get tested than nulliparous women. Whether our results reflect that multiparous women are exposed to more viral infections, but not necessarily SARS-CoV-2, or that they are in fact at higher risk of SARS-CoV-2 related to having been pregnant before, is unclear.

Obesity and gestational diabetes have been associated with severe COVID-19, but not when studying infection independent of severity^1^. We found that being overweight or obese was associated with 20–50% increased odds of test-positivity under non-universal testing, but not under universal testing. Similarly, women with gestational diabetes had 67% increased odds of test-positivity under non-universal testing, but no increased odds was seen in women under universal testing. We also saw that these background factors, as well as asthma, were associated with increased testing. Having been informed that certain conditions may be associated with severe disease could have prompted affected pregnant women to get tested. This overrepresentation of women with morbidities and obesity in tested women could induce spurious associations. No association with preeclampsia and test-positivity independent on testing strategy was found in the Swedish data.

Previous studies have highlighted that individuals of lower socioeconomic status are at higher risk of COVID-19 infection^12,13,19^. For instance, in a cohort of pregnant women in Italy, D’ambrosi et al. showed that women born outside Italy that were positive for SARS-CoV-2, were more likely to have a lower education level, to be unemployed and to live in larger families compared to women born in Italy^12^. In Sweden there was no association with education level after adjustment for age and birth region, whereas in Norway lower levels of education and unemployment was associated with test-positivity and increased likelihood of getting tested. We found that pregnant women with greater household crowding had a significantly increased odds of being test-positive, which is in line with a study of New York city residents^19^.

The only risk factor associated with COVID-19 independent of severity in the meta-analysis by Allotey et al., was being of non-white ethnicity^1^, highlighting potential ethnic and socioeconomic inequalities^20,21^. In this study, information on ethnicity was unfortunately not available. We used region of birth as a proxy for ethnicity, and found robust associations between being born in countries outside of Scandinavia and test-positivity. Similar results have been seen in Italy^12^ and Denmark^9^ where foreign-born pregnant women were more often test-positive compared to women born within each respective country. Furthermore, non-Scandinavian women in our study were less likely to get tested for SARS-CoV-2. While studies from other countries such as the United States may be influenced by differential access to health care due to financial barriers^13,21,22^, antenatal care in Sweden and Norway is free of charge and almost all women attend antenatal care. Nevertheless, whether our results are related to genetic predisposition and/or susceptibility due to comorbid conditions, factors associated with exposure, social deprivation or cultural attitudes related to the pandemic^23^ needs to be further investigated as this may lead to barriers of seeking, utilizing and getting proper access to health care.

Under non-universal testing, smoking status in early pregnancy was negatively associated with test-positivity in Sweden. Others have found that current smoking was negatively associated with hospitalization with SARS-CoV-2^8^, which was suggested to be a result of residual confounding. In our study, the protective association with smoking was not observed under universal testing, which strengthens the hypothesis that residual confounding, other underlying behavioral characteristics or test practices could affect the association. In unadjusted analyses, smokers were less likely to get tested for SARS-CoV-2, although there was no difference between smokers and non-smokers after adjustment for age, education level and region of birth, respectively.

A potential problem with studies attempting to identify risk factors for SARS-CoV-2 is collider bias^16^. When a risk factor and an outcome both affect the likelihood of being sampled, this may induce spurious associations. In COVID-19 research this bias is likely when studies are restricted to hospitalized patients, volunteers or when testing is performed only in those with symptoms under non-universal testing. We aimed to address these selections and how they may have affected previously described associations. Universal testing strategies most likely give more valid estimates of associations as these women are tested without selection and independently of underlying characteristics usually associated with the likelihood of getting tested. Women who are asymptomatic or have mild symptoms will be captured to a larger extent than those tested in a non-universal testing based on symptoms. However, as the majority of women tested under universal testing are tested when admitted for labor, women who are asymptomatic or have only mild symptoms of SARS-CoV-2 earlier on in pregnancy will not be captured. We compared characteristics of test-positive pregnant women under universal testing and non-universal testing strategies which provide valuable insights in how these different strategies may affect observed associations.

A strength of our study is the population-based data collected for up to 18 months of the pandemic. Information were from validated registers with electronically transferred data from medical records on all tested pregnant women, independent of hospital admission^5,8,24,25^. This minimizes risk of reporting bias related to maternal and neonatal morbidity because women with COVID-19 may have been more carefully investigated.

This study had a number of limitations. First, even while using the whole birthing population of pregnant women in two countries, the number of cases of SARS-CoV-2 test-positive women, especially in Norway were restricted, making power an issue for some of our adjusted analyses. Second, while our population-based findings may be generalizable to each respective country, generalizability to other countries with more markedly different testing strategies or other population background set-up may be more difficult. Third, in the beginning of the pandemic in Sweden and Norway, testing was initially focused on patients in hospitals with severe COVID-19 symptoms, and non-universal testing was only available to the broader population starting around June, 2020^18^. Consequently, the overall Swedish and Norwegian COVID-19 statistics are probably underestimated during the early months of the pandemic for those with mild symptoms^18^. Fourth, it is possible that pregnant women included in the universal testing group whom tested negative could have been infected earlier on in pregnancy but then been asymptomatic or only had mild symptoms which at that time point did not lead to testing within the non-universal testing strategy, leading to a potential differential misclassification of outcome. This in turn could lead to both an under- and overestimation of associations. Fifth, the registers wherefrom our data originates, do not include information on symptomatology.

In this registry-based study of pregnant women giving birth in Sweden and Norway during the first 18 months of the pandemic, we demonstrate that test strategy, choice of comparison group and individual background characteristics, affect who is being tested and the identification of potential risk factors for a positive test for SARS-CoV-2. Nevertheless, pregnant women born in the Middle East or Africa were consistently across all comparisons at increased odds of test-positivity. They were also less likely to be tested—suggesting that there may be difficulties of seeking, utilizing and getting proper access to health care for this group. Overall, our results have implications for surveillance, vaccine and clinical recommendations to pregnant women, both during potential coming waves of COVID-19 and future pandemics, and each country must be aware of the differences their own test policy and background characteristics may have.

## Supplementary Information

Supplementary Information.

## Data Availability

Data are available by applying to the registry owners: https://helsedata.no/soknadsveiledning/ and https://www.medscinet.com/gr/forskare.aspx.
