# Supplementary material for: The association between maternal characteristics and SARS-CoV-2 in pregnancy: a population-based registry study in Sweden and Norway
Source: Sci Rep. 2022 May 19;12:8355. doi: 10.1038/s41598-022-12395-y (PMC9120467; doi:10.1038/s41598-022-12395-y)
Supplement: Supplementary file 1 — Supplementary Information. [file 41598_2022_12395_MOESM1_ESM.docx]

**Supporting Information**

Data in Swedish and Norwegian national health registries are registered with a personal identity number (pin), which is given all citizens in Sweden and Norway for identification and public administration purposes. This pin enables linkages of individual level information across registries.

**Details of data sources and linkages**

**Sweden**

*The Swedish Pregnancy register*

Data in this study was provided through the Swedish Pregnancy Register. This quality register was initiated in 2013, and includes 92% of all births (live and stillbirths) after 22 completed gestational weeks in Sweden. Demographical, reproductive and maternal health care data, starting at the first visit to the antenatal care clinic around the ninth gestational week, are transferred from electronic medical records within 24 hours from a reported birth. From this register we used information on maternal age (years), parity (number of previous births), body mass index (kg/m^2^), smoking status (yes/no) and pre-existing co-morbidities (chronic hypertension, diabetes (type 1 and 2), asthma/lung disease, chronic kidney disease, cardiovascular disease, and prior thrombosis) based on check-boxes filled by the midwife at the first visit to the antenatal care clinic, as well as work situation (employed, unemployed, maternity leave, sick leave, student or other) and cohabitation with partner (yes/no).

*Total population register*

The Total population register, held by Statistics Sweden and initiated in 1968, contains information on all individuals residing in Sweden based on information from the Swedish Tax Agency. The register is updated monthly. From this register we used information on the mother’s country of birth and the number of people living within the mother’s household.

*Education register*

The Education Register, also held by Statistics Sweden and initiated in 1985, holds information on completed education levels which is continuously and mandatorily reported from schools and universities in Sweden. Information on immigrants’ education levels are primarily reported from questionnaires to newly arrived immigrants who are missing information in the Education register, as well as from population and housing censuses. The register is updated yearly. In Sweden, there is compulsory schooling up to 9 years. From this register completed education level in years was used.

*Swedish Register for Communicable Diseases (SmiNet)*

SmiNet is the Swedish Public Health Agency’s national register for communicable diseases. On February 1, 2020, SARS-CoV-2 was included in the Swedish Communicable Diseases Act, making it mandatory to report all laboratory-confirmed Polymerase Chain Reaction (PCR) cases within 24 hours to the register. From this register we used information on positive tests as well as the date of a positive PCR test. Ten individuals in Sweden had two positive tests. For these individuals the first positive test was used in the analyses.

**Norway**

*The Emergency Preparedness Register for COVID-19 (Beredt C19)*

Data in this study was provided through the Emergency preparedness register for COVID-19 (Beredt C19) administered by the Norwegian Institute of Public Health, according to the Health Preparedness Act §2-4. This registry was established in 2020 to give authorities up to date data for generating knowledge of the prevalence, causal relationships, and consequences of the COVID-19 epidemic in Norway. Beredt C19 includes information that has already been collected in the healthcare service, national health registries and administrative registers with information about the Norwegian population. The data subjects' right is safeguarded as they can contact the data controller for all different sources included in Beredt C19 in the usual way.

Through Beredt C19 we used data from the following sources:

*The National Population Register*

This register contains information on all persons residing in Norway. We used this information to obtain information on the woman’s exact date of birth, country of birth, and the status of her pin number (permanent or temporary)

*The Medical Birth Registry of Norway*

The Norwegian national birth registry includes information on all pregnancies ending in gestational week 12 or later from 1967 onwards. The registry is registered with the woman’s pin and includes information on live births, stillbirths, late miscarriages, and late induced abortions. Midwives register information on maternal background characteristics and health during pregnancy, in addition to information on pregnancy outcomes and neonatal health of offspring.

*Norwegian Surveillance System for Communicable Diseases*

There is mandatory reporting of selected infectious diseases to this National Health register. Reporting of all COVID-19 test is mandatory, and this register contains date of testing, test results, and the pin for each citizen.

*Statistics Norway*

Administrative data is mandatorily reported to Statistics Norway. We used information from this database on years of education completed by 2019.

| **Table S1** Dates for universal testing by delivery hospital | | | | | |
| --- | --- | --- | --- | --- | --- |
| Delivery Hospital | Universal Testing | Start 1 | Stop 1 | Start 2 | Stop 2 |
| BB Stockholm | Yes | 2020-12-18 |  |  |  |
| Borås Södra Älvsborgs sjukus | No |  |  |  |  |
| Danderyds sjukhus | No |  |  |  |  |
| Eksjö Höglandssjukhuset | No |  |  |  |  |
| Eskilstuna Mälarsjukhuset | Yes | 2020-04-02 | 2020-05-12 | 2020-11-02 |  |
| Falu lasarett | No |  |  |  |  |
| Gävle sjukhuset | Yes | 2020-04-29 |  |  |  |
| Sahlgrenska GBG | Yes | 2020-11-27 |  |  |  |
| Halmstad Hallands sjukhus | Yes | 2021-01-13 |  |  |  |
| Helsingborgs lasarett | Yes | 2020-12-15 |  |  |  |
| Huddinge Karolinska | Yes | 2020-03-25 | 2020-09-24 |  |  |
| Hudiksvalls sjukhus | Yes | 2020-04-29 |  |  |  |
| Jönköping Ryhov | No |  |  |  |  |
| Kalmar länssjukhuset | No |  |  |  |  |
| Karlskrona Blekingesjukhuset | No |  |  |  |  |
| Kristianstad Centralsjukhuset | Yes | 2020-12-14 |  |  |  |
| Linköping Universitetssjukhuset | Yes | 2021-01-25 |  |  |  |
| Lund Skånes Universitetssjukhus | Yes | 2021-01-01 |  |  |  |
| Lycksele lasarett | Yes | 2020-12-20 |  |  |  |
| Malmö Skånes Universitetssjukhus | Yes | 2021-01-01 |  |  |  |
| Norrköping Vrinnevisjukhuset | No |  |  |  |  |
| Nyköpings lasarett | Yes | 2020-04-02 | 2020-05-12 | 2020-11-02 |  |
| Skellefteå lasarett | Yes | 2021-01-25 |  |  |  |
| Skövde Skaraborgs sjukhus | No |  |  |  |  |
| Solna Karolinska Universitetssjukhuset | Yes | 2020-03-25 | 2020-09-24 |  |  |
| Stockholm Södersjukhuset | No |  |  |  |  |
| Sundsvall Länssjukhuset | No |  |  |  |  |
| Södertälje sjukhus | No |  |  |  |  |
| Trollhättan NU-sjukvården | No |  |  |  |  |
| Umeå Norrlands Universitetssjukhus | Yes | 2020-12-03 |  |  |  |
| Varberg Hallands sjukhus | Yes | 2021-01-13 |  |  |  |
| Visby lasarett | Yes | 2020-04-20 |  |  |  |
| Värnamo sjukhus | No |  |  |  |  |
| Västerviks sjukhus | Yes | 2021-01-14 |  |  |  |
| Västerås/Västmanlands sjukhus | Yes | 2020-07-15 | 2021-02-07 | 2021-02-15 |  |
| Ystads lasarett | Yes | 2021-01-18 |  |  |  |
| Örebro Universitetssjukhus | No |  |  |  |  |
| Örnsköldsviks sjukhus | No |  |  |  |  |
| Östersunds sjukhus | Yes | 2020-07-09 |  |  |  |
